# Supplementary material for: Complex‐centric proteome profiling by SEC‐SWATH‐MS
Source: Mol Syst Biol. 2019 Jan 14;15(1):e8438. doi: 10.15252/msb.20188438 (PMC6346213; doi:10.15252/msb.20188438)

Q7Z6J9_Q8NCE0
Annotated subunits: 4 Subunits with signal: 3
Max. coeluting subunits: 3 Max. completeness: 0.75

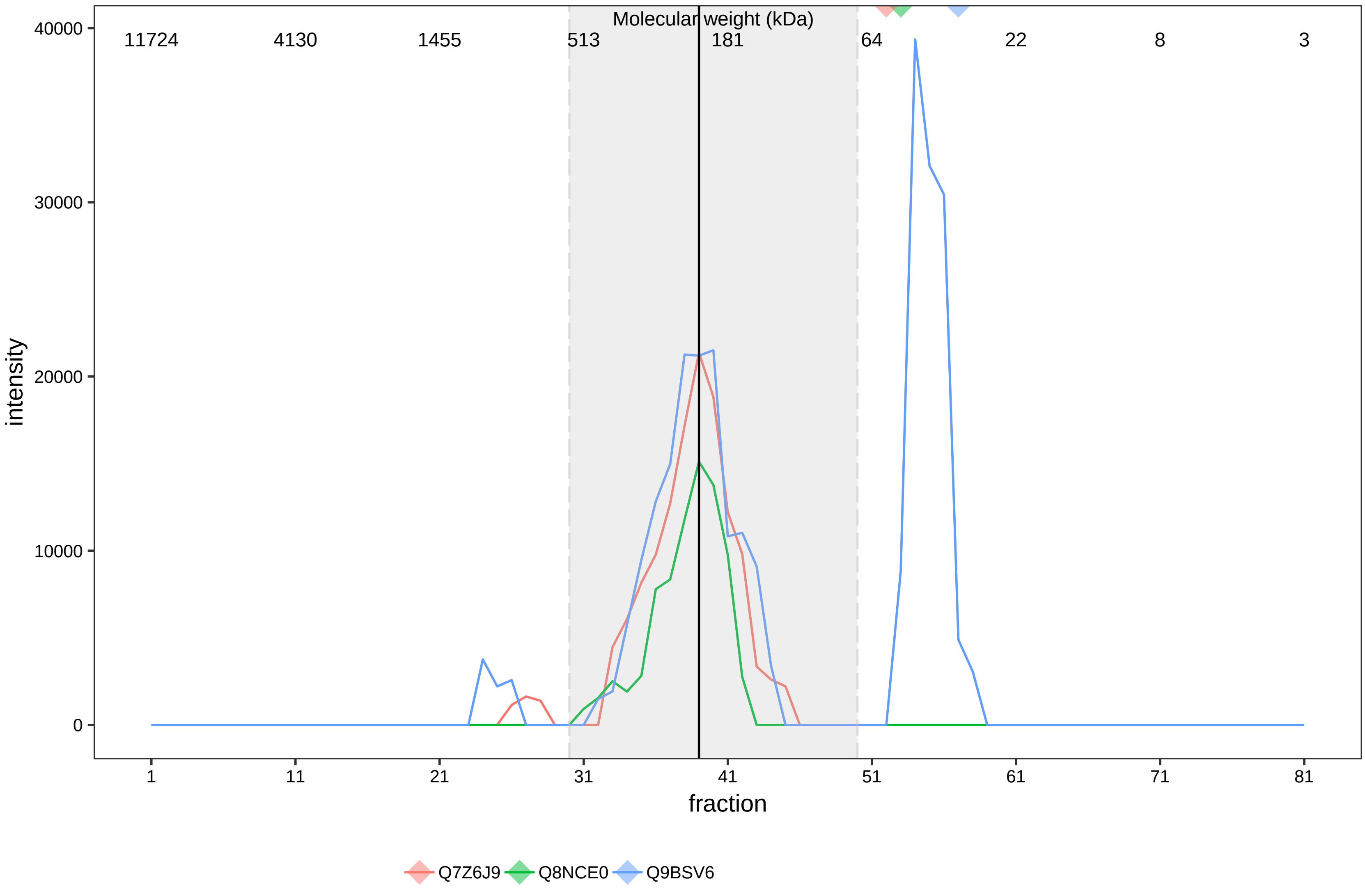

Supplement: Supplementary file 8 — Dataset EV7 [file MSB-15-e8438-s008.zip › feature_plots_string/Q7Z6J9.pdf]
